# Women’s experiences seeking informal sector abortion services in Cape Town, South Africa: a descriptive study

**DOI:** 10.1186/s12905-017-0443-6

**Published:** 2017-10-02

**Authors:** Caitlin Gerdts, Sarah Raifman, Kristen Daskilewicz, Mariette Momberg, Sarah Roberts, Jane Harries

**Affiliations:** 1 Ibis Reproductive Health, 1330 Broadway, Oakland, CA 94612 USA; 2 University of California, San Francisco, USA; 3 Advancing New Standards in Reproductive Health, University of California, 1330 Broadway, Oakland, CA 94612 USA; 4 Women’s Health Research Unit, School of Public Health and Family Medicine, Faculty of Health Sciences, University of Cape Town, Observatory, 7925 South Africa

**Keywords:** Snowball sampling, Abortion, Unsafe abortion, South Africa, Illegal abortion

## Abstract

**Background:**

In settings where abortion is legally restricted, or permitted but not widely accessible, women face significant barriers to abortion access, sometimes leading them to seek services outside legal facilities. The advent of medication abortion has further increased the prevalence of informal sector abortion. This study investigates the reasons for attempting self-induction, methods used, complications, and sources of information about informal sector abortion, and tests a specific recruitment method which could lead to improved estimates of informal sector abortion prevalence among an at-risk population.

**Methods:**

We recruited women who have sought informal sector abortion services in Cape Town, South Africa using respondent driven sampling (RDS). An initial seed recruiter was responsible for initiating recruitment using a structured coupon system. Participants completed face-to-face questionnaires, which included information about demographics, informal sector abortion seeking, and safe abortion access needs.

**Results:**

We enrolled 42 women, nearly one-third of whom reported they were sex workers. Thirty-four women (81%) reported having had one informal sector abortion within the past 5 years, 14% reported having had two, and 5% reported having had three. These women consumed home remedies, herbal mixtures from traditional healers, or tablets from an unregistered provider. Twelve sought additional care for potential warning signs of complications. Privacy and fear of mistreatment at public sector facilities were among the main reported reasons for attempting informal sector abortion. Most women (67%) cited other community members as their source of information about informal sector abortion; posted signs and fliers in public spaces also served as an important source of information.

**Conclusions:**

Women are attempting informal sector abortion because they seek privacy and fear mistreatment and stigma in health facilities. Some were unaware how or where to seek formal sector services, or believed the cost was too high. Many informal methods are ineffective and unsafe, leading to potential warning signs of complications and continued pregnancy. Sex workers may be at particular risk of unsafe abortion. Based on these results, it is essential that future studies sample women outside of the formal health sector. The use of innovative sampling methods would greatly improve our knowledge about informal sector abortion in South Africa.

**Electronic supplementary material:**

The online version of this article (10.1186/s12905-017-0443-6) contains supplementary material, which is available to authorized users.

## Background

In 1996, South Africa passed legislation called the Choice on Termination of Pregnancy Act allowing legal termination of pregnancy on request up to 12 weeks gestation, and for socioeconomic or medical reasons from 12 to 20 weeks. Beyond 20 weeks, permission of two medical practitioners is required [1]. Abortion is provided at no cost to women in public facilities. Despite the legalization of abortion, barriers to access remain [2–4]. A shortage of trained and willing providers and a lack of dedicated facilities in which to perform abortions have resulted in waiting lists that delay abortions by weeks [5, 6].

Abortion is highly stigmatized in South Africa, wait times are long, and women’s experiences with reproductive health care in both public and private sector services are often fraught with delays and mistreatment [7, 8]. This is particularly true for vulnerable groups of women, such as poor or rural women, youth, HIV positive women, or sex workers. As a result, women may resort to other methods of pregnancy termination, such as visiting an unregistered provider or self-inducing [9]. Abortions outside of the formal health system are known to occur widely in South Africa; advertisements for abortion services are posted on fliers all over public spaces in Cape Town and Johannesburg and online. The proportion of later term abortions performed in South Africa is relatively high, which has important implications for safety given that most mortality and morbidity from unsafe procedures happens in later gestational age abortions [1, 10, 11]. For the purposes of this study, we propose the term ‘*informal sector abortion’* to refer to induced abortions that occur outside of the formal health system.

Little is known about the prevalence, safety, or efficacy of informal sector abortion, women’s experiences with abortion outside the formal health system, or the impact of informal sector abortion on women’s health in South Africa [9, 12, 13]. We also know very little about the mechanisms through which women learn about informal sector abortion services in South Africa. Studies have shown that women in diverse contexts rely on friends and community members for information about abortion [14–16], and that women with strong social networks have more success in overcoming logistical barriers to accessing care [17]. Given that abortion is a right protected by law under the South African constitution, reliable information about women’s experiences with induced abortion outside of the formal health system is urgently needed in order to inform advocacy efforts and program planning aimed at improving and expanding legal abortion services in South Africa. As such, the aim of this study was to better understand women’s behavior, knowledge, and experiences seeking informal sector abortions in South Africa, as well as to test a specific recruitment method, which could lead to improved estimates of prevalence of informal sector abortion among a particularly at-risk population.

## Methods

### Formative research

Three methods are commonly used to reach hidden populations, such as women who have attempted informal sector abortion [18]. These include: snowball sampling and other forms of chain referral samples [19]; key informant sampling, which overcomes response biases by selecting especially knowledgeable respondents and asking them about others’ behavior [20]; and targeted sampling, which overcomes deficiencies in chain-referral methods by mapping the target population and recruiting a pre-specified number of subjects at sites identified by ethnographic mapping [21]. In this study, SRa conducted key informant interviews with individuals identified as knowledgeable about women’s behaviors regarding informal sector abortion in Cape Town, including providers, advocates, and researchers (see Additional file 1 for interview guide). Then, we employed the recruitment techniques, but not the analysis methods, from a specific variation of snowball sampling called respondent driven sampling (RDS) [18]. RDS differs from snowball sampling in that it enables researchers to make unbiased estimates from chain-referral sampling by weighting participants by the size of their social network during the analysis phase [22]. RDS recruitment includes the identification of a “seed” participant who is given a set number of coupons with which they can recruit their social network peers. Once a participant with a valid coupon presents to the study site, she is provided with the same number of coupons with which to enroll other members of the social network, thus resulting in a lengthy chain of participants representing the target population [18, 23]. Four elements differentiate the RDS recruitment process from the typical snow ball sampling approach: 1) use of a coupon system to document recruitment chains; 2) rationed recruitment with a specific allotment of coupons per seed; 3) collection of information on personal network size; and 4) pre-existing relationships between recruiters and recruits within recruitment chains [24].

In order to identify a potential “seed” for this study, SRa conducted 11 key informant interviews with providers, advocates, NGO staff, and researchers in November 2014 in Cape Town, South Africa. Several potential seeds were identified who maintained close connections with women who had experienced informal sector abortion in South Africa. Through referrals, we contacted three potential “seeds,” informed them about the study aims and scope, and invited them to participate in the study. Ultimately, we chose one “seed” on the basis that she felt comfortable with the task, had experience with the issue of informal sector abortion herself, and was confident that several of the women she knew, who had pursued informal sector abortion, would be willing to share their experiences.

### Recruitment

The seed distributed six recruitment coupons to women they knew. Those women who redeemed their coupon and completed the survey received six additional coupons to distribute to their respective contacts who also had experience with informal sector abortion. Each coupon had a unique identifier and included contact information for the research staff and recruits were instructed to call the phone number on the coupon to learn more about the study. Study staff, including KD and MM, provided preliminary information about the study by phone and scheduled a time to meet in person to conduct the survey. Recruits were screened for eligibility at the interview. Eligibility criteria included being age 18–50, able to speak English, and having attempted an informal abortion in the last 5 years. If participants had a valid coupon (i.e. a unique study coupon which had not been copied, forged, or previously submitted by someone else) upon arrival and provided written consent to participate in the study, the survey was then conducted. Because of the recruitment coupon techniques that we employed, and restrictions on time and budget for the project, active recruitment for the study stopped once the 16th participant was enrolled—a stopping point which could have yielded a maximum potential 96 participants.

### Survey

The survey was administered via Survey Monkey by female interviewers trained in qualitative and quantitative research at the masters’ level; survey answers were recorded on an electronic device as the participant answered each question (see Additional file 2 for survey instrument). The survey aimed to collect information on three areas of interest: 1) Information seeking behavior around obtaining informal sector abortion, 2) Existence and composition of social networks for information needs about informal sector abortion, 3) Safe abortion information and access needs of peers, close female friends, partners, and family. The survey included multiple choice and yes/no questions, with minimal open-ended questions (such as employment type), and was conducted in person in a private room at a secure facility. The specific sections of the survey included: demographic information (age, education, marital status, and employment); family planning and reproductive health history (number of live births, number of children, number of abortions, and contraceptive use); experience with informal sector abortion (number of informal sector abortions, methods used, information sources, gestational age, type of provider, reasons for not seeking legal abortion, cost, location of services, information about dosage and timing, side effects, abortion outcome, and additional treatment); and knowledge of other women’s experiences with informal sector abortion. Survey data were described with descriptive statistics, using Stata 13 (College Station, Texas).

Ethical approval was obtained from the Human Research Ethics Committee, University of Cape Town and from the Committee on Human Research at University of California, San Francisco (IRB #14–13,060). Two sets of incentives were provided to participants: participants received the first incentive (ZAR100) when they completed the survey with a valid coupon and they received an additional incentive (ZAR50) for each participant they subsequently recruited in to the study; recruiters were linked to their recruits by the identification numbers on their respective coupons. Women who were not eligible received a nominal amount (ZAR20) to cover travel expenses.

## Results

**Fig. 1:**
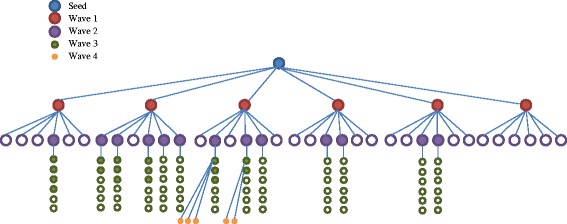
Recruitment Flow. Filled in circles are participants who completed the survey. The seed (blue) recruits wave 1 participants (red), which recruit wave 2 participants (purple), and so on

The study recruitment and interviews took place in March and April of 2015. A total of 96 coupons were distributed. Consequently, 67 women presented for screening and 43 women enrolled in the survey. The remaining 24 women were ineligible to participate: 13 had not attempted informal sector abortion, five did not present with a valid coupon, three had an abortion more than 5 years ago, three were over the age of 50 years, and two did not speak English [see Additional file 2]. One participant was later excluded from the sample of 43 because the research team was unconvinced by her recount of events, and one participant was maintained in the sample despite not having followed through with the informal sector abortion that she had sought —leaving 42 total participants Fig. 1, 41 of whom had attempted an informal sector abortion, 1 of whom had sought but ultimately not attempted an informal sector abortion. At the conclusion of data collection, 29 recruitment coupons were still in circulation. One additional recruit contacted the study staff to set up an interview, but did not attend the appointment and was unreachable by phone.

### Socio-demographic characteristics

Table 1 presents data on the socio-demographic characteristics of the study population. The average age of participants was 34 years old (SD 7.2, range 21–48 years). Approximately one-third (31%) of women had finished primary school, 45% had finished some high school, 19% had completed high school, and just under 5% had completed some tertiary education. More than half of participants were single (55%), and just over one third of participants (36%) were married or in a long-term relationship. Nearly half of our study population (45%) reported that they were unemployed at the time of the interview, while 56% of women reported full or part-time employment. A substantial minority (29%) of participants reported that they were sex workers.

**Table 1:** Socio-demographic characteristics of RDS respondents

	Mean	SD, range
Age (*n* = 42)	34.4	7.2, 21–48
Education (n = 42)	*n*	*%*
Primary	13	31
High 9–11	19	45
Matric	8	19
Tertiary	2	5
Relationship status (*n* = 42)	*n*	*%*
Single	23	55
Married/long term relationship	15	36
Divorced/Widowed	4	10
Employment (n = 42)		
No response	19	45
Sex worker	12	29
Domestic work/Cleaning	3	7
Shop or Bar lady	3	7
Peer educator or advisor	4	10
Odd jobs	1	2

Table 2 presents data on the reproductive histories of women in the sample, including use of contraception and pregnancy history. All participants had been pregnant prior to the study (mean 3.3 pregnancies (SD = 1.46, range 1–7)) and the majority of participants (88%) had given birth. On average, women had two children each (SD 1.3, range 0–5).

**Table 2:** Reproductive History of RDS respondents

	Mean	SD, range
Gravida (n = 42)	3.3	1.46, 1–7
Births (n = 42)	1.93	1.26, 0–5
Number of Children (n = 42)	1.81	1.3, 0–5
	n	%
Used contraception in last year (n = 42)	34	81
Current contraceptive use (n = 35) ^a^	28	67
Male condom	18	43
Injectables	12	29
Female condom	4	10
Female sterilization	3	7
Implant	1	2
IUD	1	2
Vasectomy	0	0
Rhythm method	0	0
Withdrawal	0	0
Ever had an abortion (n = 42)	42	100
One	34	81
Two	6	14
Three	2	5
Ever had an abortion in a facility (n = 42)	5	12
Number of informal sector abortions (n = 42)		
One	34	81
Two	6	14
Three	2	5
Went to doctor before informal sector abortion (n = 41)	6	15

^a^The percentages associated with each contraceptive method represent the number of participants out of the full sample (42) who were using that method

### Social networks of informal sector abortion

Ninety percent of women knew others in their family or community who had experiences with informal sector abortion. On average, women knew 3.7 other women (SD = 3, range 1–15) and one-third of the study participants knew four or more women who had experiences with informal sector abortion (Table 3). The majority of women (74%) told someone about their abortion attempts (after the fact); of those, most told friends (64%) or community members (15%) but rarely did women talk to family members about their experiences.

**Table 3:** Information sharing about informal sector abortion

	Mean	SD, range
Number of other women known to participants who have had informal sector abortions	3.66	*3.02, 0–15*
	*n*	*%*
Talked about abortion with others (n = 41)		
Friend	27	66
Family member	8	20
Other community members	6	15
Provider (Doctor, Pharmacist, Paramedic, Traditional healer)	3	7
Husband/partner	2	5
No one	11	27
Know others who have had informal sector abortions (n = 42)		
0	4	10
1–2	13	31
3	11	26
4–5	10	24
6 or more	4	10
Relationship to participant (n = 121)		
Friend	74	61
Family	23	19
Neighbor/community member	18	15
Other	6	5
Source of information about other women’s experiences (n = 121)		
Heard from woman herself	77	64
Heard from others	42	35
Other	2	2
Information source on informal sector abortion^a^ (n = 42)		
Community member	29	69
Sign or fliers	10	24
Family	3	7
Community group	3	7
Traditional healer	1	2
Internet	1	2
Partner	0	0
Pharmacist	0	0
Missing	1	2
Access to informal sector abortion (n = 41)		
“Very easy or Somewhat easy”	33	79
“Somewhat difficult or very difficult”	8	19
Missing	1	2

^a^Some women listed multiple information sources about backstreet abortion

The primary source of information about informal sector abortion for women in our study was other community members (67%), but nearly one quarter of women (23%) learned about informal sector providers from posted signs or fliers (Table 3). To a lesser extent, family members (7%) and community groups (5%) were also sources of information.

### Informal sector abortion experiences

All participants except one reported having had at least one informal sector abortion within the past 5 years: 81 % of participants reported one informal sector abortion in the past 5 years, 14% reported two in the past 5 years, and 5 % reported having had three in the past 5 years. Most women (80%) reported that accessing informal sector abortion services within their community was very easy. However, over half (55%) of the women said they would not recommend the experience to other women and 64% said they would not have an informal sector abortion again if they needed to terminate a pregnancy (Table 4).

**Table 4:** Characteristics of informal sector abortion experiences and providers

	Mean	SD, range
Self-reported gestational age at time of abortion-seeking (*n* = 37)	8.76	3.79, 2–18
Cost of informal sector abortion (in Rand) (n = 41)	163	146.8, 0–450
	*n*	*%*
Self-reported gestational age at time of abortion-seeking		
< =6 weeks	10	24
7–11 weeks	18	43
12 or more weeks	9	21
Missing	5	12
Method of estimating weeks pregnant		
Remembered last menstrual period	21	50
Urine pregnancy test	7	17
Ultrasound	4	10
Other	5	12
Missing	5	12
Cost of informal sector abortion		
< 50 Rand	12	29
Between 50 and 100 Rand	8	19
100–250 Rand	7	17
300 or more Rand	14	33
Missing	1	2
Why did you decide not to go to a doctor or facility?*		
Worried someone would find out	19	44
Worried about mistreatment/judgement	13	30
Wanted an abortion ASAP	5	12
Cost	2	5
Didn’t know where to go	1	2
Other	4	9
Told about possible side effects (n = 41)		
By informal sector provider	14	33
By Friend, family, community member	8	19
By Traditional healer	2	5
Missing	18	43
Experienced potential warning signs of complications (sought care for potential warning signs of complications)*		
Heavy bleeding	39 (11)	93 (26)
Cramping/abdominal pain	37 (3)	88 (7)
Dizziness	24 (2)	57 (5)
Nausea	21 (2)	50 (5)
Weakness/Fatigue	9 (0)	21 (0)
Vomiting	6 (0)	14 (0)
Fever	4 (0)	10 (0)
None	2	5
Received medical care for complications	12	28.6
Pain medication	10	24
Antibiotics	1	2
Hospitalization	2	5
Surgery	1	2
Sought additional treatment to end pregnancy		
Went to another informal provider	1	20
Went to public facility	3	60
Went to traditional healer	1	20
Would recommend informal sector abortion to others (n = 41)		
No	23	55
Yes	16	38
Not sure	2	5
Missing	1	2
Would have an informal sector abortion again (*n* = 40)		
No	27	64
Yes	7	17
Not sure	6	14
Missing	2	5

The reasons that women decided to terminate their pregnancies outside of the formal health system were primarily related to concerns about privacy and mistreatment: 44% of participants reported seeking informal sector abortions because they worried someone would find out about the abortion, and 30% reported concerns about mistreatment and stigma from providers in the formal sector. Most women (88%) had never had an abortion at a healthcare facility and few participants (15%) reported having gone to a doctor or health facility before pursuing informal sector abortion. Logistical reasons also played a role for why women sought care in the informal sector, including timeliness of services (5 women), lack of knowledge of where to seek legal services, and cost.

#### Gestational age

Half of participants reported having known their gestational age at the time of abortion based on their last missed period (50%), others learned their gestational age through over-the-counter pregnancy tests (17%), ultrasound (10%), or other methods such as recognizing pregnancy symptoms or manual assessment at a clinic or from an illegal provider (12%). The average gestational age reported by women at the time of abortion seeking was nearly 9 weeks (SD = 3.79, range 2–18). About one-fifth of participants were 12 or more weeks in the pregnancy (Table 4).

#### Reported potential warning signs of complications

More than half of the study population (57%) reported having been told about possible side effects from informal sector abortion providers (33%), friends, family or community members (19%) and traditional healers (5%) (Table 3). About 93% of women reported experiencing “heavy bleeding” during the abortion, and 11 women (26%) sought care for “heavy bleeding” from a doctor or clinic (Table 4). Many women also reported cramping and abdominal pain (88%), dizziness (57%), and nausea (50%). One woman had an infection following her informal sector abortion and received antibiotics at a public health facility. When their attempts failed, five women sought additional treatment to end their pregnancies from a different informal provider, public facilities, and a traditional healer.

#### Method of informal abortion

Table 5 presents detailed information about the methods used by study participants to terminate pregnancies. The majority of participants consumed home remedies made of substances they bought from the chemist or a local shop (Table 5). One woman did not ultimately have an informal sector abortion and therefore did not report an abortion method type. The substances ingested by women included Dutch remedies (57%); herbal remedies, such as Stametta, a cure-all product consisting of aloe, ascorbic acid, and magnesium sulfate (33%); abrasive substances like steel wool (14%); laxatives (19%); household cleaning agents (14%); alcohol (19%); and other miscellaneous medications (19%). One of the 13 women who ingested herbal mixtures from a traditional healer was given instructions to boil abrasive substances mixed with newspaper. Seven women who bought tablets from unregistered providers described the tablets as varying in shape, size and color. The number of tablets provided to women ranged from two to six and were presumably a combination of misoprostol and pain medication, based on the visual descriptions women provided. None of the women in the sample reported using instrumentation to induce abortion. About 39% of the women who went to traditional healers, 38% of women who took home remedies, and 29% of women who obtained tablets from an unregistered provider received additional medical treatment following their abortion attempts.

**Table 5:** Substances used by RDS participants to induce abortion

N (%)	Where substance obtained	Categories of type of substance		additional medical treatment received (N (%)
13 (31%)	Traditional healer	Herbal mixtures of unknown content, ranging from 100 to 750 ml. One woman was given instruction to boil abrasive substance mixed with newspaper and swallow it with herbal mixture.		5 (38.5%)
21 (50%)^a^	Home remedies purchased from chemist or shop	Herbal remedy (e.g. Stametta)	7 (33%)	8 (38%)
		Dutch remedies *Haarlamans/Versterkdruppels/Essence of life* *Vornokroy* *Helmins drops* *Potassium permanganate*	12 (57%) 7 (33%) 2 (10%) 2 (10%) 1 (5%)	
		Abrasive substances (e.g. steel wool mixed with oro crush)	3 (14%)	
		Laxatives (e.g. Castor Oil and Epsom salts)	4 (19%)	
		Household cleansing agents	3 (14%)’	
		Bleach	1 (5%)	
		Ammonia based items (e.g. Handy Andy and Jeyes Fluid)	2 (10%)	
		Alcohol (e.g. Brandy and Stout)	4 (19%)	
		Miscellaneous tablets *ARV medication* *Hypertension medication* *Zifozonke*	4 (19%) 1 (5%) 1 (5%) 2 (10%)	
7 (17%)	Illegal provider	Miscellaneous tablets of varying shape, size and color (likely misoprostol)		2 (28.6%)

^a^ multiple responses allowed, percentages may add to >100%

## Discussion

While not generalizable to a broader population, this study presents unique insight into women’s experiences seeking abortion in Cape Town, South Africa. Some women in our study did not know where or how to seek abortion services within the formal health system, and two women believed the cost would be lower outside the formal health system—despite the fact that public sector facilities are required to offer services for free. That thirty-percent of women in our study cited seeking an informal sector abortion because of concerns about privacy, mistreatment and stigma from providers is a striking commentary on the perceptions of quality of abortion care in the formal sector. In a sample population consisting of many sex workers, mistrust of the formal health care system is not unexpected; sex workers seeking abortion in Cape Town likely face a double stigmatization related to abortion and their work. For this reason, this is a sub-population which is often overlooked and under-represented in studies about abortion. Until now, data on women’s experiences with abortion services in South Africa have come primarily from women seeking abortion care within the formal sector [9, 13]. While these studies have indeed pointed to problems within public sector abortion services in South Africa, it is likely that their data overestimate the quality of abortion services given women’s tendency to express high levels of satisfaction with abortion with little variation [5, 6]. Our data suggest that at least some women are bypassing formal sector services entirely because of concerns over provider attitudes and service provision.

The widespread use of herbs and home remedies is somewhat surprising given current thinking with respect to the role of misoprostol in increasing access to safe abortion in restrictive settings [25, 26]. The use of unproven and potentially less safe substances to induce abortion at home is, however, consistent with data from the United States where women also cited barriers to accessing formal sector services and concerns over quality of care at abortion clinics for seeking abortion outside of a clinic setting [27]. The rate of potential warning signs of complications reported was high; more than a quarter of the sample reported seeking additional care for heavy bleeding. The similarity across findings from the United States and South Africa, two settings where legal grounds for abortion are broad but where multiple barriers to access exist, may suggest that the role of misoprostol in increasing access to safe abortion is moderated by legal settings. Perhaps, where abortion is illegal, regulations on pharmacy access to misoprostol are less strict and black market availability of medications for abortion is enabled, and where abortion is legal but difficult to access, medications for abortion may be more highly regulated leaving women with fewer options for safe informal sector abortion. More research is needed to better understand the role that abortion medications play in increasing access to safe abortion in diverse legal settings.

Our study does have some important limitations to consider. The data do not provide generalizable results about informal sector abortion and related morbidity and mortality outcomes, due to the fact that the sample size is relatively small and the study population comprised mostly female sex workers. It is possible that a largely sex worker sample is more socially networked than the larger population of women attempting informal sector abortion. We also note that seeking an informal sector abortion differs from other behaviors studied through snowball sampling techniques in that, unlike men having sex with men or injection drug use, abortion is not typically an ongoing or recurring behavior. However, in spite of this distinction, it does seem that snowball sampling (and specifically RDS recruitment strategies) are able to identify women who engage in the stigmatized behavior of seeking informal sector abortion. Finally, though we did successfully employ RDS recruitment strategies (i.e. the coupon system for recruiting and tracking study participants), it is important to note that a full RDS analysis was not completed, and further research should be conducted to test the feasibility of applying an RDS approach to measuring the incidence of informal sector abortion.

Our decision to compensate with a slightly higher number of coupons per recruiter than is typical for RDS recruitment [24] could have influenced recruiters to suggest participation to those who were in-eligible and contributed to the high rate of ineligibility among coupon holders. Verification of the eligibility criteria that participants have “had an informal sector abortion”, however, often proved complex, primarily due to a lack of clarity in terminology referring to informal sector abortion, and the constantly evolving nature of terms like “illegal” and “unsafe” in reference to abortion. In addition, a few participants provided inconsistent or vague stories, which further complicated the interviewers’ ability to accurately determine participant eligibility. Some individual stories came across as scripted or pre-prepared. Interviewers at times suspected that participants may have been ‘coached’ by recruiters with respect to the type of questions researchers would ask, and may have prepared the responses they needed to provide in order to appear eligible for the study. In these few challenging cases it was left to the interviewers’ discretion whether or not to enroll the recruits in the study. It is possible that recruiters and potential participants may have been motivated by the study’s incentive structure to appear eligible even if they were not. While this incentive structure is standard in relation to other research studies conducted in the Cape Town area [28–30], for future studies we will carefully review the amount and frequency of incentives offered and make additional efforts to communicate the importance of actual eligibility to recruiters.

Finally, it is unclear whether the substances ingested in attempt to terminate pregnancy, such as steel wool or corrosives, could have induced pelvic bleeding specifically as opposed to gastric ulceration and consequent bleeding in the gastro-intestinal system. The survey did not precisely quantify or define “heavy bleeding”; therefore, the “heavy bleeding” reported here reflects the language women used to describe their experiences.

## Conclusions

This is the only known study to directly sample women outside of the formal health system in an effort to understand informal sector abortion experiences in South Africa. Our results shed important light on the concerns that women have, specifically with public sector abortion services, which may lead them to seek abortions outside of the formal sector.

## Additional files

Additional file 1: Interview Guide. This is the interview guide used to interview key informants. (DOCX 33 kb)

Additional file 2: Survey Instrument. This is the survey instrument used to survey participants. (PDF 450 kb)

## Abbreviations

IRB: Institutional review board
IUD: Intrauterine device
RDS: Respondent Driven Sampling
SD: Standard deviation
ZAR: South African Rand

## References

[1] Harries J, Orner P, Gabriel M, Mitchell E (2007). Delays in seeking an abortion until the second trimester: a qualitative study in South Africa. Reprod Health.

[2] Harries J, Cooper D, Strebel A, Colvin CJ (2014). Conscientious objection and its impact on abortion service provision in South Africa: a qualitative study. Reprod Health.

[3] Jewkes RK, Gumede T, Westaway MS, Dickson K, Brown H, Rees H (2005). Why are women still aborting outside designated facilities in metropolitan South Africa?. BJOG Int J Obstet Gynaecol.

[4] Trueman KA, Magwentshu M (2013). Abortion in a progressive legal environment: the need for vigilance in protecting and promoting access to safe abortion services in South Africa. Am J Public Health.

[5] Grossman D, Constant D, Lince N, Alblas M, Blanchard K, Harries J (2011). Surgical and medical second trimester abortion in South Africa: a cross-sectional study. BMC Health Serv Res.

[6] Harries J, Lince N, Constant D, Hargey A, Grossman D (2012). The challenges of offering public second trimester abortion services in South Africa: health care providers' perspectives. J Biosoc Sci.

[7] Brown H, Hofmeyr GJ, Nikodem VC, Smith H, Garner P (2007). Promoting childbirth companions in South Africa: a randomised pilot study. BMC Med.

[8] Chadwick RJ, Cooper D, Harries J (2014). Narratives of distress about birth in South African public maternity settings: a qualitative study. Midwifery.

[9] Constant DGDLNHJ (2014). Self-induction of abortion among women acessing second-trimester abortion services in the public sector, Western Cape Province, South Africa: an exploratory study. S Afr Med J.

[10] DOH. Termination of Pregnancy Update Cumulative Statistics through 2004. In: Edited by Health SADo. Pretoria: South African Department of Health; 2005.

[11] Harris LH, Grossman D (2011). Confronting the challenge of unsafe second-trimester abortion. Int J Gynaecol Obstet.

[12] Gerdts C, DePiñeres T, Hajri S, Harries J, Hossain A, Puri M, Vohra D, Foster DG. Denial of abortion in legal settings. J Fam Plann Reprod Health Care. 2014;13:jfprhc-2014. 10.1136/jfprhc-2014-100999 PMC4501171 25511805

[13] Harries J, Gerdts C, Momberg M, Greene Foster D (2015). An exploratory study of what happens to women who are denied abortions in Cape Town, South Africa. Reprod Health.

[14] Banerjee SK, Andersen KL, Buchanan RM, Warvadekar J (2012). Woman-centered research on access to safe abortion services and implications for behavioral change communication interventions: a cross-sectional study of women in Bihar and Jharkhand, India. BMC Public Health.

[15] Grossman D, Holt K, Pena M, Lara D, Veatch M, Cordova D, Gold M, Winikoff B, Blanchard K (2010). Self-induction of abortion among women in the United States. Reprod Health Matters.

[16] Rossier C (2007). Abortion: an open secret? Abortion and social network involvement in Burkina Faso. Reproductive Health Matters.

[17] Ostrach B, Cheyney M (2014). Navigating Social and Institutional Obstacles: Low-Income Women Seeking Abortion. Qual Health Res.

[18] Heckathorn DD (1997). Respondent-driven sampling: a new approach to the study of hidden populations. Soc Probl.

[19] Goodman L (1961). Snowball Sampling. Ann Math Stat.

[20] Deaux EC, Callaghan JW (1985). Key informant versus self-report estimates of health behavior. Eval Rev.

[21] Watters JB (1989). Targeted sampling: Options for the study of hidden populations. Soc Probl.

[22] Heckathorn DD (2011). Snowball Versus Respondent-Driven Sampling. Sociol Methodol.

[23] Johnston LG, Sabin K (2010). Sampling hard-to-reach populations with respondent driven sampling. Methodol Innov Online.

[24] Magnani R, Sabin K, Saidel T, Heckathorn D (2005). Review of sampling hard-to-reach and hidden populations for HIV surveillance. AIDS.

[25] Fernandez MM, Coeytaux F, de Leon RG, Harrison DL (2009). Assessing the global availability of misoprostol. Int J Gynaecol Obstet.

[26] Winikoff B, Sheldon W. Use of medicines changing the face of abortion. Int Perspect Sex Reprod Health. 2012:164–6. 10.1363/3816412 23018138

[27] Zurbriggen R, Keefe-Oates B, Gerdts C. Accompaniment of second-trimester abortions: the model of the feminist Socorrista network of Argentina. Contraception. 2017. 10.1016/j.contraception.2017.07.170 28801052

[28] Townsend L, Jewkes R, Mathews C, Johnston LG, Flisher AJ, Zembe Y, Chopra M (2011). HIV risk behaviours and their relationship to intimate partner violence (IPV) among men who have multiple female sexual partners in Cape Town, South Africa. AIDS Behav.

[29] Kimani SM, Watt MH, Merli MG, Skinner D, Myers B, Pieterse D, MacFarlane JC, Meade CS (2014). Respondent driven sampling is an effective method for engaging methamphetamine users in HIV prevention research in South Africa. Drug Alcohol Depend.

[30] Chopra M, Townsend L, Johnston L, Mathews C, Tomlinson M, O'Bra H, Kendall C (2009). Estimating HIV prevalence and risk behaviors among high-risk heterosexual men with multiple sex partners: use of respondent-driven sampling. J Acquir Immune Defic Syndr.

