# Supplementary material for: Women’s experiences seeking informal sector abortion services in Cape Town, South Africa: a descriptive study
Source: BMC Womens Health. 2017 Oct 2;17:95. doi: 10.1186/s12905-017-0443-6 (PMC5625615; doi:10.1186/s12905-017-0443-6)
Supplement: Supplementary file 1 — Interview Guide. This is the interview guide used to interview key informants. (DOCX 33 kb) [file 12905_2017_443_MOESM1_ESM.docx]

**RDS Formative Interview Guide**

**UCSF/UCT**

**2013**

Guideline for In-depth interviews with women

Introduction

- Introduce yourself and thank the participant for agreeing to meet you.
- Explain the purpose of the study and how the interview will be conducted (why s/he was selected, recording, what the information will be used for).
- Before you start the interview, obtain verbal informed consent.

**Key reminders for Interviewer**

- The interview guide is only a guide. Try to cover all of the issues but there is considerable flexibility for the participant to tell you what s/he feels is important, and for you to find out about other interesting/related issues.
- Each question is followed by possible probes and issues to cover. You should only use these as examples or to generate ideas, not as a checklist.
- The ordering of the questions is intentional but you don’t have to follow it if the respondent begins discussing something of interest which hasn’t yet been covered; just return to the earlier question afterwards.
- Try to build trust and rapport with the respondent throughout the interview.
- Probe where necessary and clarify vague or contradictory information. Use prompts like:
  - Can you tell me more about that?
  - What do you mean exactly?
  - Am I right in thinking that…?
  - What do you think about…?
  - Do you mean that…?
  - Earlier you told me…but here you seem to be saying…Can you clarify this?

**1. Participant Background**

| **Topic Focus** | **Core questions** | **Additional questions or prompts** |
| --- | --- | --- |
| **Personal Background** | - Could you please tell me about yourself? | - Age, marital status, number of living children, ethnicity, occupation, level of education, number of abortions (lifetime) |

**2. Decision making process**

| **Decision making** | - Can you tell me was happening in your life at the time of the pregnancy? - Can you describe the circumstances that led to your decision to have an abortion? | - When did you first discover that you were pregnant? How did feel about the pregnancy at that time? - What circumstances initially compelled you to decide to terminate the pregnancy? - Do you remember talking to anyone about getting an abortion? If so, who did you talk to? What advice were you looking for? What information did you receive? - Was the decision to abort influenced by the advice given by others you may have spoken with? If so, who influenced your decision? - Was there anyone else involved in this discussion about terminating the pregnancy? - Who had the final say on the decision? - What considerations did you have to think about in making this decision? - Were there any difficulties (impediments) that you faced in making this decision? - How long did it take from the initial decision making to actually seeking an abortion/visiting a provider? |
| --- | --- | --- |

**3. Abortion seeking behavior**

|  | Can you describe the process you went through to seek an abortion? *( please ask about all the process and all places visited)* | Where did you go first? How did you find out that place? What were you told? What information advice you were given? *(see probes below depending on if she sought abortion from a provider or attempted to self-induce using medications or traditional methods)* |
| --- | --- | --- |
| **Provider** | ***If sought abortion from any kind of provider***  Tell me what happened when you went to this provider. [*try to capture the chronology of events, concerns or dilemmas that were occurring, and what emotions were involved]*  Was the abortion successful? If no, why not? | - - - Without giving me anyone’s name, what kind of person did you seek an abortion from/where did you go?     - How did you make the decision that this was trustworthy provider?     - What happened?     - How did you feel about what happened? (*probe to see if she felt it was a safe place/if she felt safe, probe to see if she is aware about government approved place*)     - How much did the abortion cost?     - Were you satisfied with the outcome? What concerns do you remember having? Did you get any advice about seeking medical care after the aobrtion? What was the advice?     - Did you experience any complications following the abortion?   If so, what kind of complications did you face?   - - - Did you seek medical or other care after the abortion? What medical care did you seek and why?     - Is there anything you wish that you had known before going to this provider? |
| **Self-induction** | ***If not visited elsewhere for an abortion:***  If yes, can you tell me about your decision making process to end the pregnancy yourself?  Can you tell me about your experience trying to end the pregnancy yourself?  Was the abortion successful? | - Did you seek any information or advice about it? - From where? From whom? What information advice you were given? How did you know that this information was accurate? (*ask separately for each source of advice*) - How did you decide what to do to end the pregnancy yourself? - How did you try to end the pregnancy yourself? - How did you feel about what happened?   ***(if used medications)***   - - What medication/s did you use?   - Without giving me anyone’s name, where did you get the medication?   - How much did the medications cost?   - What, if any, directions did you get about using the medications?   - Without giving me anyone’s name, where did you get directions about using the drug?     - Did you experience any complications following the abortion?   If so, what kind of complications did you face?   - Did you seek medical or other care after the abortion? - What medical care did you seek and why? |

**5. Social Network**

|  | Of the women you know, how many might have terminated a pregnancy at some time or the other? *(Probe: Think of your friends, relatives, and neighbors)* | - Without telling me their names, how do you know they may have terminated a pregnancy? How well do you know them? |
| --- | --- | --- |
|  | Do you know or spend time with other women who have had the same kind of abortion you had (or any abortion that was not performed at a registered abortion clinic or public health facility)? | - How many women do you know who have had the same kind of abortion you had (or any abortion that was not performed at a registered abortion clinic or public health facility)? Can you tell me about your relationship with each one of them. *[Probes: is she family, friend, co-worker, neighbor, etc.; how long have you known her? How often do you see her? How often do you talk with her? How do you know that she terminated a pregnancy?]* - How many of these women have you seen in the past month? - Do these women know each other?   - **[If yes]** what is their relationship like? Do they ever discuss the abortions they had with each other? Do they share information about seeking this kind of abortion with other friends/family? |
| We are considering doing a future study about women who have had the kinds of abortions that you have had. In the study, women would be contacted by a friend, or someone in their social network, and asked to participate in a 20-30 minute interviews. We would not collect any identifying information from women (no names, phone numbers, addresses, signatures, nothing) Women would get paid for their participation in the study. We are not asking you to agree to participate in this future study, but we would like to ask your opinions about how we could best conduct such a study.   - Would you (or your peers) be willing to participate in this survey? - Why or why not? - Would you be willing to recruit your peers into this study? - If you were asked to contact three peers who have had the kind of abortion that you have had and encourage them to participate in the study, how many of them do you think would actually want to participate? - What might prevent your peers from participating in the survey? - How would you encourage a friend to join the study? Especially one who is reluctant? - Can you suggest anyone who would be especially good at recruiting women for this study (for example, someone who is a source of information about this kind of abortion, or someone who just knows a lot of women who have had this kind of abortion)? - Can you suggest other people/organizations we should talk to as we are planning this study? | | |

**6. Knowledge of the law and advice for others seeking abortion**

|  | Tell me what you know about the abortion law in South Africa? | - What do you know about the abortion law in South Africa? Do you know on what conditions a can woman can have legal abortion in South Africa? *(Be sure to note carefully any misinformation or incorrect information respondent might have about the existing law)* |
| --- | --- | --- |
|  | If someone approached you about an unintended pregnancy, someone who is experiencing the same situation that you had and they asked you what they should do, what would you say to them? Why? |  |
