# Supplementary material for: Women’s experiences seeking informal sector abortion services in Cape Town, South Africa: a descriptive study
Source: BMC Womens Health. 2017 Oct 2;17:95. doi: 10.1186/s12905-017-0443-6 (PMC5625615; doi:10.1186/s12905-017-0443-6)
Supplement: Supplementary file 2 — Survey Instrument. This is the survey instrument used to survey participants. (PDF 450 kb) [file 12905_2017_443_MOESM2_ESM.pdf]

RDS Study

Section 1: Demographic Inf...

1. Participant Details

Screening ID Number

Participant ID Number

Participant's RDS Coupon Number

2. Interview date

DD MM YYYY

Date / /

Instructions to interviewer: ask all questions and responses as written. Follow prompts for instructions on probing.

Take note of the following codes:

66= participant refusal

88= participant does not know answer

3. How old are you?

*4. What is the highest level of education you completed?

- no school
- primary school
- high school(9-11)
- matric
- tertiary
- Undisclosed

5. What is your marital status?

- single
- married/in long term relationship
- divorced
- widowed
- Undisclosed

6. Are you employed?

(If NO, Skip ---> Q8)

7. What kind of paid work do you do?
(Probe as necessary)

Section 2: FP & RH His...

8. Have you used a contraceptive method in the past year?
(If NO, skip ---> Q11)

9. Are you currently using a contraceptive method?
(If NO, skip ---> Q11)

10. What method(s) are you currently using?
(Tick all that apply)

- ☐ Female sterilization (tubes tied)
- ☐ Pill
- ☐ IUD
- ☐ Injectables
- ☐ Implant
- ☐ Male condom
- ☐ Female condom
- ☐ Male sterilization (vasectomy)
- ☐ Rhythm method
- ☐ Withdrawal
- ☐ Other (please specify)

Now I would like to ask about **all** the live births and pregnancies you have had during your life

11. How many times in your life have you been pregnant, including any pregnancies that were not carried to term?

12. How many times have you given birth?

13. How many children do you have? (i.e. how many children are you currently raising?)

14. How many abortions have you had?

(Interviewer: check total number of pregnancies add up - If 0 previous abortions, end of interview)

15. Have you ever had an abortion in a healthcare facility?

Section 3: Experience with...

Now I'd like to ask you about your experiences with abortion outside of the health system (i.e. "backstreet abortion")

Probe: by this I mean,

- Where you might have contacted a non-licensed abortion provider online or through a friend?
- Responded to an ad for abortion that you saw on a lamp post; in a taxi/train; train station/taxi rank or a pamphlet?
- Taken/eaten/drank medications or other substances on your own to try to end a pregnancy
- Gone to see a sangoma or traditional healer to end a pregnancy

16. Have you ever had a backstreet abortion?

(If NO/Undisclosed previous Backstreet Abortions, end of interview)

17. For how many pregnancies have you attempted/had a backstreet abortion?

(If 0 previous Backstreet Abortions, end of interview)

In reference to the *first time* you had a backstreet abortion:

18. What method(s) was used to (attempt) to end the pregnancy?

- ☐ Something you drank (e.g. Stametta)
- ☐ Bought medication from an unregistered provider
- ☐ Bought herbs or tablets from a pharmacy or shop
- ☐ Got something from a herbalist
- ☐ Sangoma (witch doctor)
- ☐ Other (please specify)

19. When you first considered ending your pregnancy with backstreet abortion, who/what did you use to find out information about it? ***(Click all that apply)***

- ☐ Family member
- ☐ Partner
- ☐ Other community member
- ☐ Pharmacist
- ☐ Community group
- ☐ Internet search
- ☐ Posted sign
- ☐ Other (please specify)

(If she did not have a second (attempted) backstreet abortion, skip ---> Q24).

In reference to the *<u>second time</u>* you had a backstreet abortion:

20. What method(s) was used to (attempt) to end the pregnancy?

- ☐ Something you drank (e.g. Stametta)
- ☐ Bought medication from an unregistered provider
- ☐ Bought herbs or tablets from a pharmacy or shop
- ☐ Got something from a herbalist
- ☐ Sangoma (witch doctor)
- ☐ Other (please specify)

21. When you first considered ending your pregnancy with backstreet abortion, who/what did you use to find out information about it? ***(Click all that apply)***

- ☐ Family member
- ☐ Partner
- ☐ Other community member
- ☐ Pharmacist
- ☐ Community group
- ☐ Internet search
- ☐ Posted sign
- ☐ Other (please specify)

(If she did not have a third (attempted) backstreet abortion, skip ---> Q24).

In reference to the *third time* you had a backstreet abortion:

22. What method(s) was used to (attempt) to end the pregnancy?

- ☐ Something you drank (e.g. Stametta)
- ☐ Bought medication from an unregistered provider
- ☐ Bought herbs or tablets from a pharmacy or shop
- ☐ Got something from a herbalist
- ☐ Sangoma (witch doctor)
- ☐ Other (please specify)

23. When you first considered ending your pregnancy with backstreet abortion, who/what did you use to find out information about it? ***(Click all that apply)***

- ☐ Family member
- ☐ Partner
- ☐ Other community member
- ☐ Pharmacist
- ☐ Community group
- ☐ Internet search
- ☐ Posted sign
- ☐ Other (please specify)

IF Backstreet Abortion ***USED MORE THAN ONCE***:
Now I'd like to ask you some questions about the **last time** you had a Backstreet Abortion.

IF Backstreet Abortion ***USED ONLY ONCE***:
Now I'd like to ask you some questions about **the time** you had a Backstreet Abortion

24. Did you know how many weeks pregnant you were when you had a Backstreet Abortion?
(If No/undisclosed, skip ---> Q28)

25. How many weeks pregnant were you?

26. How did you know how many weeks pregnant you were?

- I remembered my last missed period
- Ultrasound
- Other (please specify)

27. How certain were you about how many weeks pregnant you were?

- Very certain
- Somewhat certain
- Somewhat uncertain
- Very uncertain

28. Before you attempted/had the backstreet abortion, did you go to a doctor or health facility for help with ending the same pregnancy?
(If YES, skip ---> Q30)

29. Why did you decide not to go to a doctor or health facility for help in terminating your pregnancy?

- Too expensive
- Didn’t know where to go
- Worried someone would find out
- Worried about mistreatment/stigma from providers at health facility
- Other (please specify)

30. Why did you not have your pregnancy terminated by the doctor or health provider you visited?

- Too expensive
- Provider not available
- Past gestational age
- Other medical reason
- Other (please specify)

31. When you went for a backstreet abortion, were you ever given tablets to end your pregnancy?
(If NO/Undisclosed, skip ---> Q37)

32. What was the name of the medication(s) were you given?
(Click all that apply)

- ☐ Misoprostol alone
- ☐ Mifepristone and Misoprostol
- ☐ Don't know
- ☐ Other (please specify)

33. How many tablets were you given?

34. What did the tablets look like?
(Note size; colour; shape etc.)

35. Were you given information about how many to take and when to take them?

36. How were you told to take the tablets?

- ○ To swallow
- ○ Place under tongue or in cheek
- ○ Into my vagina (private parts)
- ○ Into my behind (rectal)
- ○ Other (please specify)

37. How much did you pay for the backstreet abortion?

38. Where did you obtain the backstreet abortion or, if self-induced, supplies for the backstreet abortion?

- ○ Pharmacist
- ○ Nurse or doctor
- ○ Community group
- ○ By mail through the internet
- ○ Backstreet abortion provider
- ○ Other (please specify)

39. When you obtained Backstreet Abortion, were you told about any possible side effects or complications you might experience?
(If NO/Not Sure, skip ---> Q42)

40. What side effects or complications were you told about?
(List all mentioned)

41. Who gave you this information?

- Backstreet provider
- Friend or family member
- Other (please specify)

42. Did you seek out any (more) information about side effects, complications, dosage, or timing from any other source?
(If NO/Undisclosed, skip ---> Q44)

43. Where did you get this additional information from?
(Click all that apply)

- Pharmacist
- Friend or family member
- Doctor or nurse
- Internet
- Hotline
- Community organization
- Other (please specify)

44. What side effects or complications did you experience?
(Click all that apply)

- ☐ Cramping/abdominal pain
- ☐ Nausea
- ☐ Dizziness
- ☐ Fever
- ☐ Heavy bleeding
- ☐ None *(--->skip Q47)*
- ☐ Other (please specify)

45. For which of these side effects or complications did you go to a doctor or clinic?
(Click all that apply)

- ☐ Cramping/abdominal pain
- ☐ Nausea
- ☐ Dizziness
- ☐ Fever
- ☐ Heavy bleeding
- ☐ None
- ☐ Other (please specify)

46. What kind of medical care did you receive?
(Click all that apply)

- ☐ No intervention
- ☐ Pain medication
- ☐ Antibiotics
- ☐ Hospitalization
- ☐ Surgery
- ☐ Another abortion
- ☐ Other (please specify)

47. Did you/the provider you went to successfully end your pregnancy without needing any other treatment?
(If Yes, skip ---> Q51)

48. Did you seek other treatment to help terminate the pregnancy after the backstreet abortion did not work?
(If YES, skip ---> Q50)

49. Why did you choose not to seek other treatment to help terminate the pregnancy after the backstreet abortion did not work?

- Too expensive
- Didn't know where to go
- Worried someone would find out
- Worried about mistreatment/stigma from providers at health facility
- Decided to keep the pregnancy
- Other (please specify)

50. What kind of treatment did you receive to help terminate the pregnancy after the backstreet abortion was not successful?

- Returned to same backstreet provider
- Went to different backstreet provider
- An abortion from private clinic
- An abortion from a public clinic
- Self-induction
- Other (please specify)

51. Would you recommend Backstreet Abortion to another person who wants to end a pregnancy and/or needs to end a pregnancy?

52. In general, how difficult do you think it would be for someone in your community to obtain a Backstreet Abortion if they needed it?

- Very easy
- Somewhat easy
- Somewhat difficult
- Very difficult

53. Have you talked about your backstreet abortion with anyone?
(If NO/Undisclosed, skip ---> Q55)

54. Who have you talked to?
(Click all that apply and elaborate)

	Answer
Husband/partner	
Family member	
Friend	
Other community members	

Please elaborate each response

55. If you needed to end another pregnancy in the future, would you have a backstreet abortion again?

Section 4: Recruitment

As I explained at the beginning, this study aims to talk to women who have had an abortion outside of the formal health system (Backstreet Abortion). You were recommended to us because you've had such an abortion and because you knows other women who have also had backstreet abortions. I would now like to ask you some questions about each person you know who has had a Backstreet Abortion and is **over the age of 18**: You can decide whom you want to recruit, and you do not need to recruit anyone or talk about the study with anyone with whom you don't feel comfortable discussing the topic of Backstreet Abortion.

56. Do you know other people in your family or community who have had backstreet abortions?

- YES
- NO
- Not Sure

57. How many people who have had backstreet abortions do you know?

PERSON 1

58. a) What is her relationship to you?

Family member, please specify

Friend

Other, specify

59. a) How do you know that she used a backstreet abortion or another method to end a pregnancy?

- She told me
- Someone else told me
- Other (please specify)

PERSON 2

60. b) What is her relationship to you?

Family member, please specify

Friend

Other, specify

61. b) How do you know that she used a backstreet abortion or another method to end a pregnancy?

- She told me
- Someone else told me
- Other (please specify)

PERSON 3

62. c) What is her relationship to you?

Family member, please specify

Friend

Other, specify

63. c) How do you know that she used a backstreet abortion or another method to end a pregnancy?

- She told me
- Someone else told me
- Other (please specify)

PERSON 4

64. d) What is her relationship to you?

Family member, please specify

Friend

Other, specify

65. d) What is her relationship to you?

Family member, please specify

Friend

Other, specify

PERSON 5

66. e) What is her relationship to you?

Family member, please specify

Friend

Other, specify

67. e) What is her relationship to you?

Family member, please specify

Friend

Other, specify

PERSON 6

68. f) What is her relationship to you?

Family member, please specify

Friend

Other, specify

69. f) What is her relationship to you?

Family member, please specify

Friend

Other, specify

We have now come to the end of the survey. Thank you so much for your time.
